# Knockdown of Thymidine Kinase 1 Suppresses Cell Proliferation, Invasion, Migration, and Epithelial–Mesenchymal Transition in Thyroid Carcinoma Cells

**DOI:** 10.3389/fonc.2019.01475

**Published:** 2020-01-29

**Authors:** Chang Liu, Jian Wang, Li Zhao, Hui He, Pan Zhao, Zheng Peng, Feiyuan Liu, Juan Chen, Weiqing Wu, Guangsuo Wang, Fajin Dong

**Affiliations:** ^1^Clinical Medical Research Center, First Affiliated Hospital of Southern University of Science and Technology, Second Clinical College of Jinan University, Shenzhen, China; ^2^Central Lab, Dalian Municipal Central Hospital, Dalian, China; ^3^Department of Thoracic Surgery, First Affiliated Hospital of Southern University of Science and Technology, Second Clinical College of Jinan University, Shenzhen, China; ^4^Department of Health Management, First Affiliated Hospital of Southern University of Science and Technology, Second Clinical College of Jinan University, Shenzhen, China; ^5^Department of Medical Research, Shenzhen Shekou People's Hospital, Shenzhen, China; ^6^Department of Ultrasound, First Affiliated Hospital of Southern University of Science and Technology, Second Clinical College of Jinan University, Shenzhen, China

**Keywords:** thyroid carcinoma, thymidine kinase 1, thyroid nodules, progression, miR-34a-5p

## Abstract

Patients with advanced thyroid carcinoma have poor prognosis with low overall survival. Unfortunately, the underlying mechanisms of thyroid carcinoma progression remain unclear. The elevated expression of thymidine kinase 1 (TK1) has been implicated in the progression of thyroid carcinoma, while the role of TK1 in thyroid carcinoma progression has not been explored. The present study aimed to determine the role TK1 in the progression of thyroid cancer and to explore the underlying molecular mechanisms. In this study, it was found that serum TK1 levels were markedly increased in the patients with thyroid nodules. Further online data mining showed that TK1 expression was upregulated in thyroid carcinoma tissues, and higher expression of TK1 was correlated with shorter disease-free survival of patients with thyroid carcinoma. Silencing of TK1 suppressed cell proliferation, invasion, migration, and epithelial–mesenchymal transition, and also induced cell apoptosis in the thyroid carcinoma cell lines. Animal studies showed that TK1 knockdown inhibited *in vivo* tumor growth of thyroid carcinoma cells. Importantly, miR-34a-5p was found to be downregulated in the thyroid carcinoma cells. Furthermore, miR-34a-5p targeted the 3′ untranslated region of TK1 and suppressed the expression of TK1 in thyroid carcinoma cell lines. In summary, first, these results demonstrated the upregulation of TK1 in thyroid nodules and thyroid carcinoma tissues; second, TK1 promoted thyroid carcinoma cell proliferation, invasion, and migration; lastly, TK1 was negatively regulated by miR-34a-5p. Our study may provide novel insights into the role of TK1 in regulating thyroid carcinoma progression.

## Introduction

Thyroid carcinoma is one of the most common human malignancies, and the incidence rate of thyroid carcinoma is expected to gradually increase (1). Based on the characteristics of histology, thyroid carcinoma can be divided into four types including anaplastic, follicular, medullary, and papillary thyroid carcinoma (2). Papillary thyroid cancer is the main type of this malignancy and accounts for more than 80% of all cases (2). The thyroid carcinoma at the early stage is commonly curable; however, patients with advanced thyroid carcinoma have poor prognosis with relatively low 5-years survival rates (3, 4). In this regard, it is important to elucidate the molecular mechanisms of thyroid carcinoma development and to develop novel diagnostic and therapeutic strategies for thyroid carcinoma.

Thymidine kinase 1 (TK1) is an important regulatory factor in modulating cell cycle. During different stages of cell cycle, the activities of TK1 are increased at the late G1 phase and reached maximum levels at the late S phase (5, 6). Dysregulation of TK1 has been shown to be associated with the progression of human malignancies. Yu et al. screened a total of 56,178 human subjects in the southeast of China and proposed that serum TK1 was a potential biomarker for the early discovery of human subjects having the risk to process into malignancy (7). Wei et al. showed that TK1 overexpression was correlated with the poor prognosis of patients with lung cancer (8). Wang et al. demonstrated that determination of TK1 expression using immunohistology could improve the overall prediction of prognosis of ovarian cancer patients (9). Knockdown of TK1 inhibited the progression of pancreatic cancer cell via targeting E2F1-TK1-P21 axis (10). In the thyroid cancer, high levels of TK1 were correlated with the advanced clinical stage of patients with thyroid carcinoma (11). Unfortunately, the molecular mechanisms of TK1 in regulating thyroid carcinoma progression have not been explored yet.

MicroRNAs (miRNAs) are an abundant class of short (18–24 nt), endogenous non-coding RNAs that act as regulators at the transcriptional or post-transcriptional level of gene expression. MiRNAs can affect essential cellular processes including cell growth, cell differentiation, and apoptosis, which are closely correlated to carcinogenesis. Aberrant expression of miRNAs has been reported in various types of cancers including thyroid carcinoma. For example, plasma miR-346, miR-10a-5p, and miR-34a-5p were elevated in papillary thyroid carcinoma, which could be used as important biomarkers for the progression of thyroid carcinoma (12).

In this study, we identified the upregulation of TK1 in the serum of patients with thyroid nodules. The *in vitro* functional studies showed that TK1 silencing suppressed thyroid cancer cell proliferation, invasion, migration, epithelial–mesenchymal transition (EMT) and induced cell apoptosis. Furthermore, the upregulation of TK1 in the thyroid cancer may be related to the downregulation the tumor-suppressive miR-34a-5p.

## Materials and Methods

### Clinical Samples

The serum samples were collected from 1,112 subjects who underwent the physical examination at First Affiliated Hospital of Southern University of Science and Technology, Second Clinical College of Jinan University between 2015 and 2018. Among the subjects, 431 patients were positive for thyroid nodules by ultrasound examination, and 681 patients were negative for thyroid nodules. The protein levels of TK1 in the serum were detected using the enzyme-linked immunosorbent assay (ELISA) assay kit (#ab223595, Abcam, Cambridge, USA). All the experimental protocols were approved by the Ethics Committee of the First Affiliated Hospital of Southern University of Science and Technology, and all the patients signed the written informed consent.

### Cell Lines and Cell Culture

The normal human primary thyroid follicular epithelial cells (Nthy-ori 3-1, #90011609) and thyroid carcinoma cell line (TPC-1, #SCC147) were obtained from Merck (Darmstadt, USA). The thyroid carcinoma cell lines (BC-PAP, #ACC273) were obtained from the German Collection of Microorganisms and Cell Cultures (Braunschweig, Germany). The cells were cultured in RMPI-1640 medium (Sigma-Aldrich, St. Louis, USA) supplemented with 10% fetal bovine serum (FBS; #10100154, Life Technologies, Waltham, USA) and were kept in a humid atmosphere of 5% (*v*/*v*) CO_2_ and 95% (*v*/*v*) air at 37°C.

### Synthesis of Small Interfering RNAs and miRNAs, Cell Transfections

The small interfering RNAs (siRNAs) that silencing TK1 (TK1 siRNA#1 and #2) or the scrambled negative control (si-NC) were designed and synthesized by Ribobio (Guangzhou, China). The miRNA mimics for miR-34a-5p and the respective mimics NC were purchased from Thermo Fisher Scientific (Waltham, USA). The thyroid carcinoma cell transfections with these siRNAs or miRNAs were performed using Lipofectamine 2000 reagent (#11668030, Invitrogen, Carlsbad, USA) according to the manufacturer's protocol, and the transfected cells were collected after 24 h of transfection for further study.

### Quantitative Real-Time PCR

Extraction of total RNAs from tissues or cells was performed using Trizol reagent (#15596018, Invitrogen) according to the manufacturer's protocol. For TK1 messenger RNA (mRNA) detection, RNAs were reversely transcribed into complementary DNA (cDNA) using the first-strand cDNA synthesis kit (#K1621, Thermo Fisher Scientific); for miR-34a-5p detection, RNAs were reversely transcribed into cDNA using the NCode miRNA first-strand cDNA synthesis kit (#MIRC10, Invitrogen). Real-time PCR was performed on an ABI7900 PCR system (Applied Biosystems, Foster City, USA) using SYBR Green PCR Master Mix (#RR820A, Takara, Dalian, China). GAPDH and U6 were used as internal control for TK1 and miR-34a-5p expression, respectively. The fold change between different groups was determined using comparative Ct method.

### Western Blot

Extraction of proteins from tissues or cells was performed using radio-immunoprecipitation assay buffer (#P0013B, Beyotime, Beijing, China) supplied with the protease inhibitor cocktail (ST506, Beyotime). The protein concentrations were determined using the bicinchoninic acid method (#23252, Thermo Fisher Scientific). Equal amount of 30 μg proteins was separated by sodium dodecyl sulfate–polyacrylamide gel electrophoresis followed by transferring to polyvinylidene fluoride membranes (#IPVH08100, Sigma-Aldrich). After incubating with 1.5% non-fat milk in the Tris-buffered saline Tween, the membranes were incubated with corresponding primary antibodies including TK1 (1:1,000; #8960, Cell Signaling Technology, Danvers, USA), active caspase-3 (1:1,000; #9661, Cell Signaling Technology), active caspase-9 (1:1,000; #7237, Cell Signaling Technology), vimentin (1:1,000) (5,741, Cell Signaling Technology), N-cadherin (1:1,000; #14215, Cell Signaling Technology), E-cadherin (1:1,000; #14472, Cell Signaling Technology), and β-actin (1:1,000; #3700, Cell Signaling Technology). After incubating with above primary antibodies overnight at 4°C, membranes were then further probed against the respective horseradish-conjugated secondary antibodies (1:2,000; #7074, #7076, Cell Signaling Technology) at room temperature for 2 h. Protein detection was performed using the enhanced chemiluminescence kit (#15159, Thermo Fisher Scientific) according to the manufacturer's protocol. β-Actin served as the loading control.

### Cell Counting Kit-8 and Colony Formation Assays

Cell proliferation was measured by cell counting kit-8 (CCK-8) assay (#C0039, Beyotime) in thyroid carcinoma cells at 0, 24, 48, and 72 h after transfection. Briefly, the transfected thyroid cells were incubated with 10 μl CCK-8 reagent for 1 h at 37°C. The cell proliferation values were determined by measuring optical density values at 450 nm.

Cell growth was measured by colony formation assay. Briefly, the transfected thyroid carcinoma cells were seeded into a six-well plate at a density of 500 cells per well, after growing for 10 days, the cells were fixed with methanol for 10 min and stained with 0.1% crystal violet (#C0121, Beyotime) for 10 min. The number of colonies was counted under a light microscope (TE2000, Nikon, Tokyo, Japan).

### Transwell Invasion Assay

Thyroid carcinoma cell invasion was measured by Transwell invasion assay. Briefly, the Matri-gel (#E6909, Sigma-Aldrich) was coated on the 8-μm pore size membrane Transwell inserts (#140629, Thermo Fisher Scientific), and the coated inserted were placed into the wells of a 24-well plate (#142475, Thermo Fisher Scientific). The transfected thyroid carcinoma cells were seeded onto the upper chamber filled with FBS-free RMPI-1640 medium, while the bottom chamber was filled with RMPI-1640 medium supplied with 10% FBS. After incubation for 24 h, the non-invaded carcinoma cells were removed from the upper surface of the membrane, and cells in the lower surface of the membrane were fixed with methanol for 20 min and stained with 0.5% crystal violet for 15 min. The number of invaded cells was counted under a microscope by randomly selecting five fields.

### Wound Healing Assay

Thyroid carcinoma cell migration was determined by wounding healing assay. Briefly, the transfected thyroid carcinoma cells were seeded onto the six-well plates (#140675, Thermo Fisher Scientific) and cultured for 24 h. The wound was created on the monolayer cells using a sterile 200-μl tip (#94410313, Thermo Fisher Scientific), and the cells were further cultured for 24 h. The wound width was measured at 0 and 24 h, respectively. The percentage of wound closure was calculated by (wound width at 24 h—wound width at 0 h)/wound width at 0 h.

### Flow Cytometry

The cell apoptosis of thyroid carcinoma cells was detected using Annexin V-fluorescein isothiocyanate/propidium iodide (PI) apoptosis detection kit (#V13241, Thermo Fisher Scientific). Briefly, the transfected thyroid carcinoma cells were trypsinized and collected, and the cells were incubated with Annexin V-fluorescein isothiocyanate and PI in binding buffer for 10 min. The stained cells were then analyzed using a BD FACSCanto II flow cytometer (BD Biosciences, San Jose, USA).

### Caspase-3 Activity Assay

The caspase-3 activity of the transfected thyroid carcinoma cells was determined using a caspase-3 activity assay kit (#5723, Cell Signaling Technology) according to the manufacturer's protocol.

### *In vivo* Tumor Growth Assay

A total of 12 male BALB/nude mice (6–8 weeks old) were obtained from Guangzhou Laboratory Animal Center (Guangzhou, China). All animal experiments were approved by the Animal Ethics Committee of First Affiliated Hospital of Southern University of Science and Technology. TPC-1 cells (5 × 10^6^ cells) with stably expressing TK1 shRNA (sh_TK1) or scrambled negative control shRNA (sh_NC) were subcutaneously injected into the right flank of the nude mice and six animals in each group. After injection of carcinoma cells, the tumor volume of the nude mice was measured every 7 days for 42 days. At the end of the experiments, the mice were killed, and the tumor tissues were collected for further analysis.

### Dual-Luciferase Reporter Assay

To construct the reporter vectors, the 3′ untranslated region (UTR) of TK1 containing the putative binding sites of miR-34a-5p was amplified by PCR and cloned into downstream of the luciferase gene of the pGL3 vector (#E1751, Promega, Madison, USA). The mutant reporter vectors were generated by mutating three nucleotides in the binding region. Thyroid carcinoma cells were cotransfected with reporter vectors and miRNAs using Lipofectamine 2000 reagent (Invitrogen). At 24 h after transfection, luciferase activity in the thyroid carcinoma cells was determined using the Dual-Luciferase Reporter Assay System (#E1910, Promega).

### Statistical Analysis

All data analysis was performed using GraphPad Prism (Version 5.0; GraphPad Software, La Jolla, USA). Summary data are presented as the mean ± standard deviation. Significant differences between different groups were evaluated using Student's *t* test or one-way ANOVA followed by Bonferroni's *post hoc* test. Statistical significance was set at *P* < 0.05.

## Results

### TK1 Was Upregulated in Serum From Patients With Thyroid Nodules and Was Upregulated in the Thyroid Carcinoma Tissues

**Figure 1:**
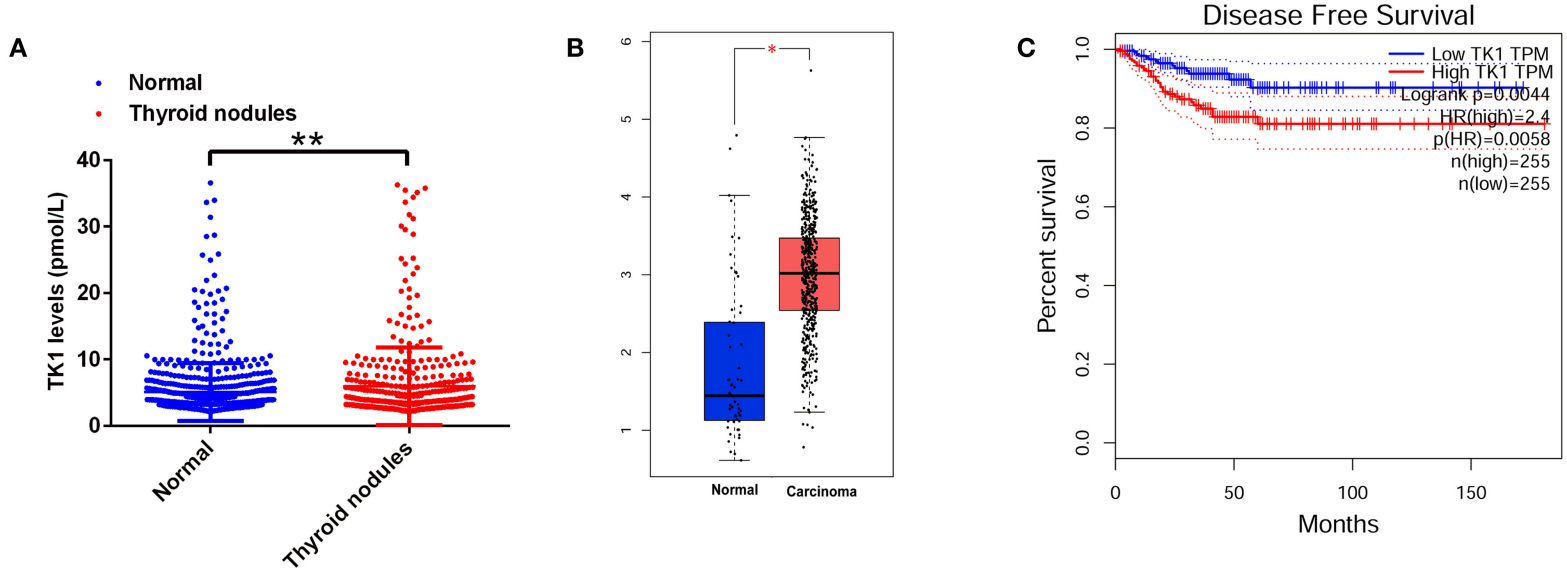
Thymidine kinase 1 (TK1) was upregulated in patients with thyroid nodules and thyroid carcinoma. **(A)** The serum TK1 levels of normal subjects and subjects with thyroid nodules were detected by ELISA assay. **(B)** Online database [The Cancer Genome Atlas (TCGA)] analysis of the TK1 expression levels in the thyroid tissues from normal subjects and thyroid carcinoma patients. **(C)** The disease-free survival of the thyroid carcinoma patients as stratified by TK1 expression levels. *P < 0.05 and **P < 0.01.

We first analyzed the serum TK1 protein levels from the subjects who underwent physical examination in our hospital and found that serum TK1 levels were significantly higher in the subjects with thyroid nodules compared to the normal subjects (Figure 1A). A further analysis using data mining tool (http://gepia.cancer-pku.cn/) showed that TK1 was markedly upregulated in the thyroid carcinoma tissues when compared to normal thyroid tissues (Figure 1B). In addition, patients with higher expression of TK1 had poorer disease-free survival when compared to patients with lower expression of TK1 (Figure 1C).

### TK1 Knockdown Suppressed Thyroid Carcinoma Cell Proliferation and Induced Cell Apoptosis

**Figure 2:**
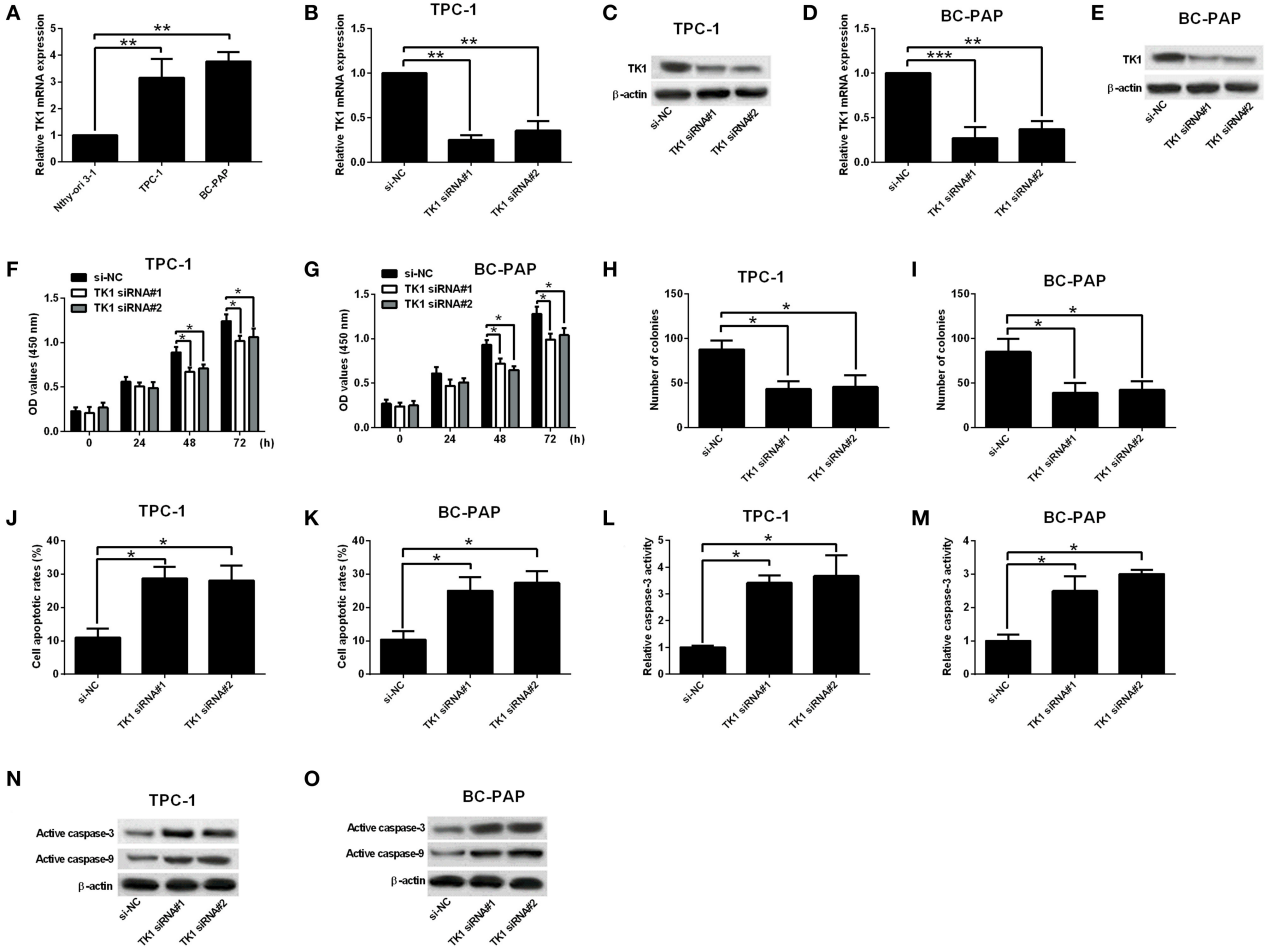
Thymidine kinase 1 (TK1) knockdown suppressed thyroid carcinoma cell proliferation and induced cell apoptosis. **(A)** Quantitative real-time PCR (qRT-PCR) analysis of TK1 messenger RNA (mRNA) expression level in normal thyroid follicular epithelial cells and thyroid carcinoma cells (TPC-1 and BC-PAP). **(B–E)** qRT-PCR and Western blot analysis of TK1 mRNA and protein expression levels in TPC-1 and BC-PAP cells after scrambled siRNA (si-NC) or TK1 siRNAs (TK1 siRNA#1 or #2) transfections. **(F,G)** Cells proliferation by CCK-8 assay was determined in TPC-1 and BC-PAP cells, respectively. **(H,I)** Colony formation ability was assessed in TPC-1 and BC-PAP cells, respectively. **(J,K)** Flow cytometry analysis was used to detect the cell apoptotic rates in TPC-1 and BC-PAP cells, respectively. **(L,M)** Caspase-3 activity assay was used to determine the capsase-3 activity of TPC-1 and BC-PAP cells, respectively. **(N,O)** Protein expression levels of active caspase-3 and caspase-9 in TPC-1 and BC-PAP cells were detected by Western blot, respectively. *N* = 3; *P < 0.05, **P < 0.01, and ***P < 0.001.

The mRNA expression of TK1 in carcinoma cells as well as in normal thyroid follicular epithelial cells was investigated. TK1 was upregulated in carcinoma cells when compared to normal thyroid follicular epithelial cells (Figure 2A). To determine whether the knockdown of TK1 could reserve the aggressiveness of carcinoma cells, TPC-1 and BC-PAP cells were transfected with scrambled siRNA or TK1 siRNAs. TK1 siRNA transfection significantly repressed mRNA and protein expression levels of TK1 in TPC-1 and BC-PAP cells when compared to scrambled siRNA transfection (Figures 2B–E). CCK-8 and colony formation assays showed that TK1 knockdown suppressed the TPC-1 and BC-PAP cell proliferation (Figures 2F,G) and colony formation ability (Figures 2H,I). Flow cytometry and caspase-3 activity analysis showed that TK1 knockdown increased cell apoptotic rates (Figures 2J,K) and caspase-3 activity of TPC-1 and BC-PAP cells (Figures 2L,M). The Western blot assay showed that TK1 knockdown increased the protein levels of active caspase-3 and caspase-9 in TPC-1 and BC-PAP cells (Figures 2N,O).

### TK1 Knockdown Suppressed Thyroid Carcinoma Cell Invasion, Migration, and Epithelial–Mesenchymal Transition

**Figure 3:**
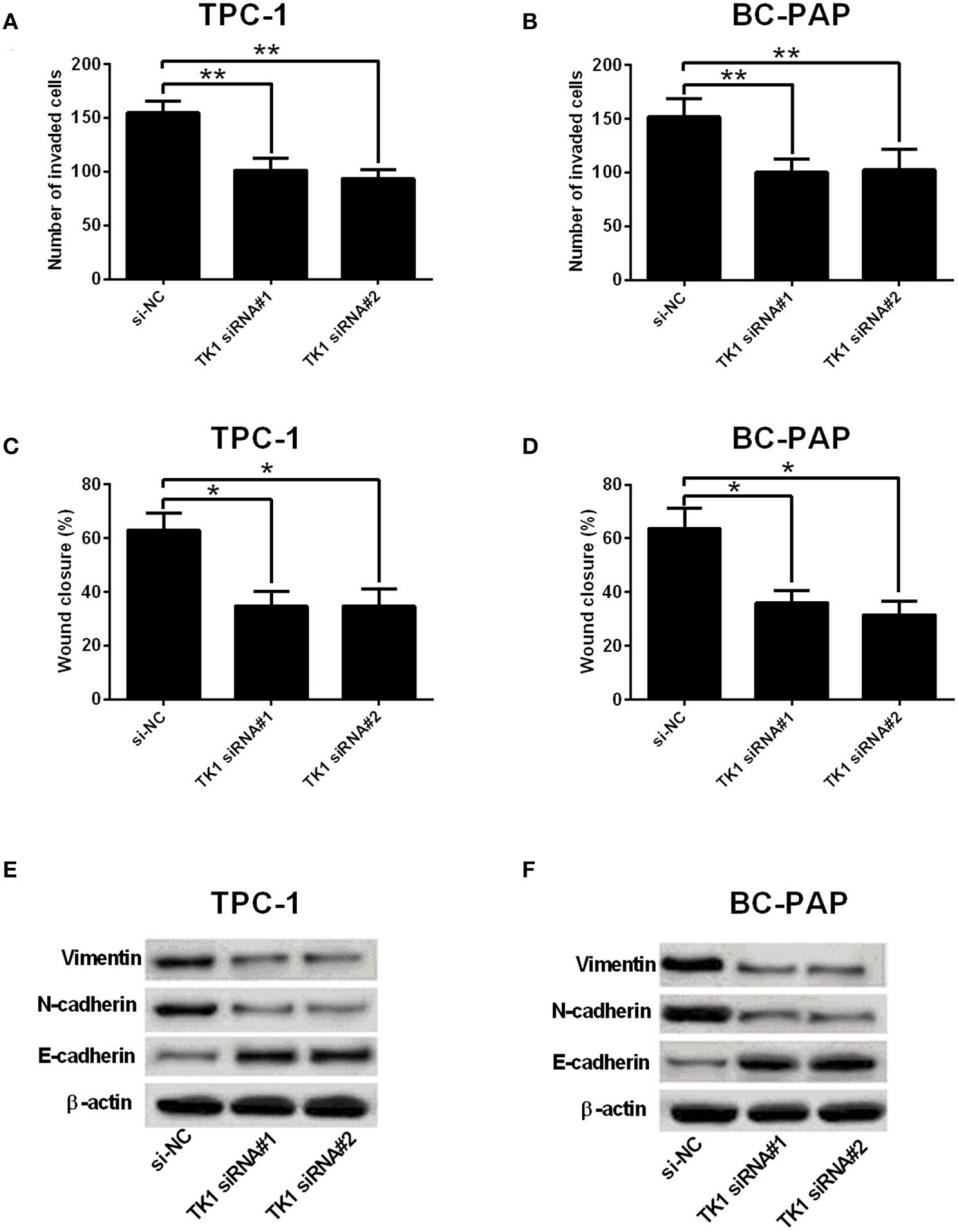
Thymidine kinase 1 (TK1) knockdown suppressed thyroid carcinoma cell invasion, migration, and EMT. **(A,B)** Transwell invasion assay was used to investigate the invasion ability of TPC-1 and BC-PAP cells transfected with scrambled siRNA (si-NC) or TK1 siRNAs (TK1 siRNA#1 or #2), respectively. **(C,D)** The migration ability of TPC-1 and BC-PAP cells transfected with scrambled siRNA (si-NC) or TK1 siRNAs (TK1 siRNA#1 or #2) was assessed by wound healing assay, respectively. **(E,F)** Protein expression levels of epithelial–mesenchymal transition (EMT)-related markers vimentin, N-cadherin, and E-cadherin in TPC-1 and BC-PAP cells transfected with scrambled siRNA (si-NC) or TK1 siRNAs (TK1 siRNA#1 or #2) were detected by Western blot. *N* = 3; *P < 0.05 and **P < 0.01.

A further analysis of cell invasion and migration using Transwell invasion assay and wound healing assay, respectively, showed that TK1 knockdown markedly suppressed TPC-1 and BC-PAP cell invasion (Figures 3A,B) and migration (Figures 3C,D). In addition, it was found that TK1 knockdown suppressed the protein levels of EMT-related markers vimentin and N-cadherin but increased the protein levels of E-cadherin (Figures 3E,F).

### TK1 Knockdown Suppressed *in vivo* Tumor Growth of Thyroid Carcinoma Cells

**Figure 4:**
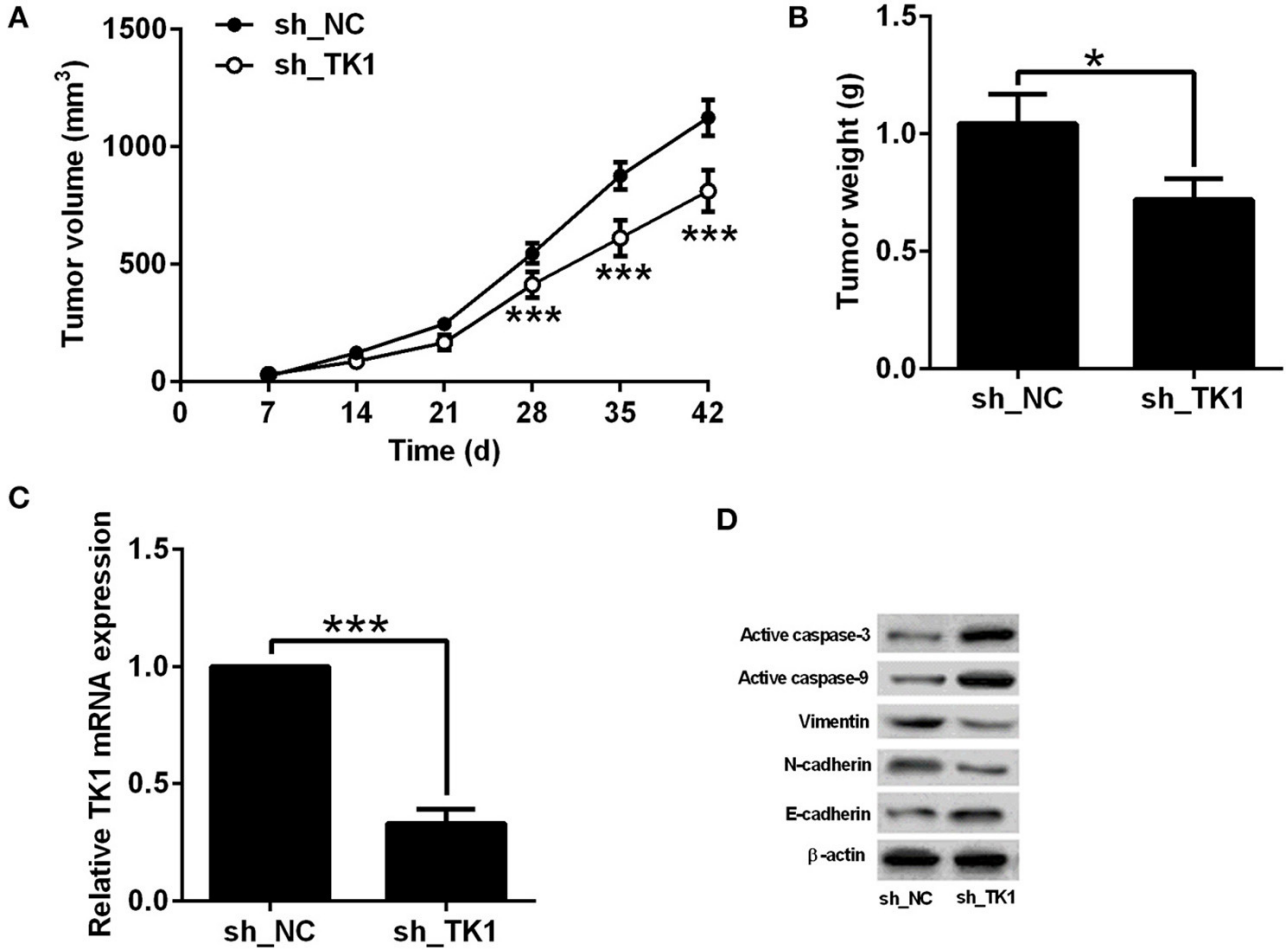
Thymidine kinase 1 (TK1) knockdown suppressed *in vivo* tumor growth of thyroid carcinoma cells. **(A)** *In vivo* tumor growth of TPC-1 cells from sh_NC group and sh_TK1 group. **(B)** Weight of the tumor tissues from sh_NC and sh_TK1 group. **(C)** qRT-PCR analysis of TK1 mRNA expression levels in tumor tissues from sh_NC and sh_TK1 groups. **(D)** Western blot analysis of active caspase-3, caspase-9, vimentin, N-cadherin, and E-cadherin protein expression levels of the tumor tissues from sh_NC and sh_TK1. *N* = 6; *P < 0.05 and ***P < 0.001.

The effects of TK1 knockdown on the *in vivo* tumor growth of TPC-1 were evaluated in nude mice xenografts model. As shown in Figure 4A, TK1 knockdown reduced the tumor volume in the nude mice; consistently, the weight of dissected tumor tissue was significantly lower in the h_TK1 group when compared to the sh_NC group (Figure 4B). The quantitative real-time PCR analysis showed that TK1 was downregulated in the tumor tissues from the sh_TK1 group when compared to the sh_NC group (Figure 4C). In addition, TK1 knockdown increased the protein levels of active caspase-3, caspase-9, and E-cadherin, but decreased the protein levels of vimentin and N-cadherin (Figure 4D).

### TK1 Expression Was Regulated by miR-34a-5p in Thyroid Carcinoma Cells

**Figure 5:**
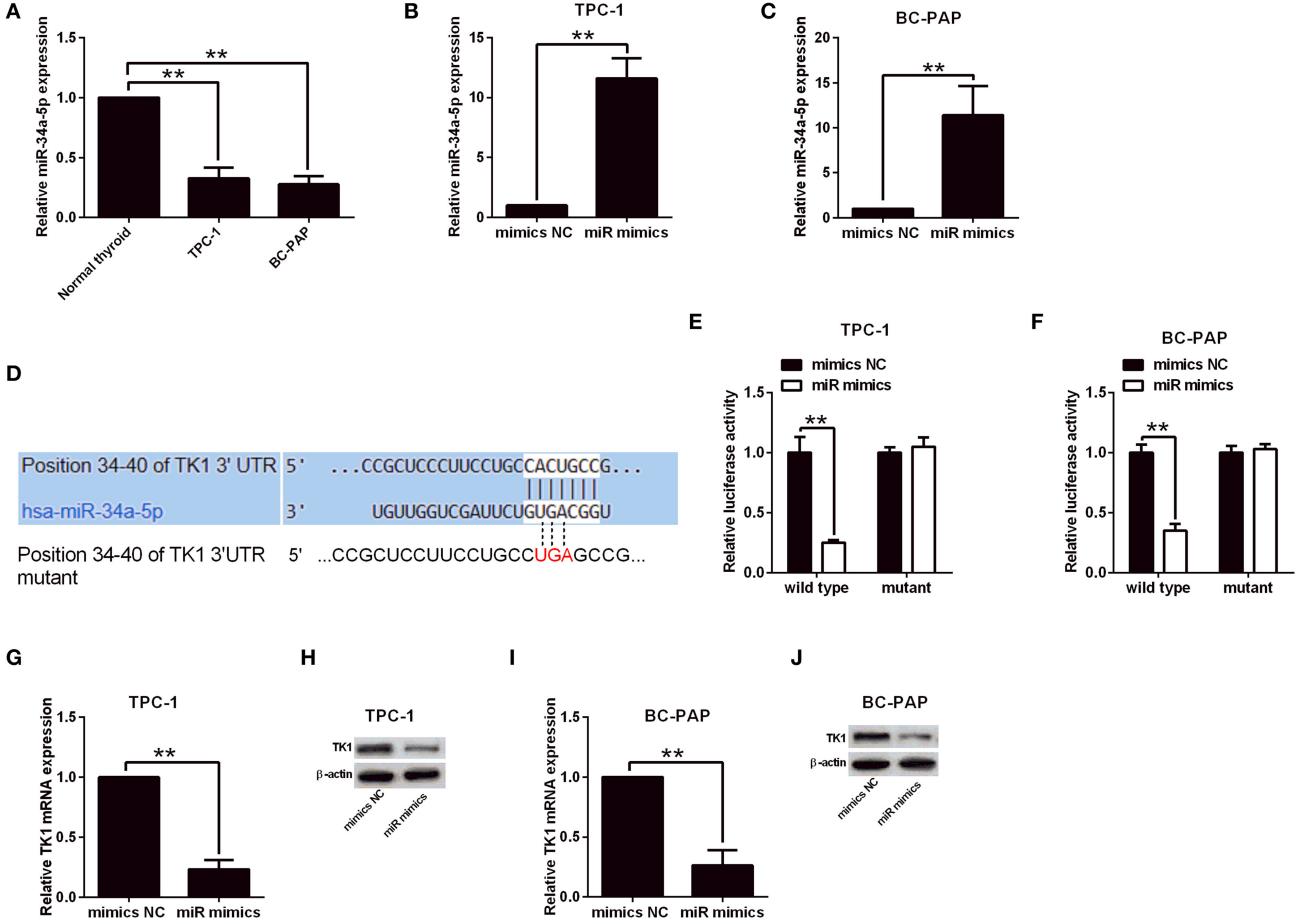
Thymidine kinase 1 (TK1) expression was regulated by miR-34a-5p in thyroid carcinoma cells. **(A)** Quantitative real-time PCR (qRT-PCR) analysis of miR-34a-5p expression level in normal thyroid follicular epithelial cells, TPC-1 and BC-PAP cells. **(B,C)** MiR-34a-5p expression in TPC-1 and BC-PAP cells with mimics NC or miR mimics transfection, respectively, by qRT-PCR analysis. **(D)** Predicted binding sites between TK1 3′UTR and miR-34a-5p by TargetScan tool. **(E,F)** Luciferase reporter assay was used to investigate luciferase activity of wild and mutant TPC-1 and BC-PAP cells with miRNAs and luciferase reporter vectors cotransfection, respectively. **(G–J)** qRT-PCR and Western blot were used to detect TK1 mRNA and protein expression levels in TPC-1 and BC-PAP cells with mimics NC or miR mimics transfection, respectively. *N* = 3; **P < 0.01.

To determine the factors that contribute to the upregulation of TK1 in thyroid carcinoma, the StarBase tool (http://starbase.sysu.edu.cn/index.php) was used to predict miRNAs that could regulate TK1 expression. Among the predicted miRNAs, miR-34a-5p was selected for examination, and it was downregulated in the TPC-1 and BC-PAP cells when compared to normal thyroid follicular epithelial cells (Figure 5A). The overexpression of miR-34a-5p was achieved by transfecting TPC-1 and BC-PAP cells with miR-34a-5p mimics (Figures 5B,C). Furthermore, the luciferase reporter assay was performed to determine the interaction between miR-34a-5p and TK1. The putative binding sites between TK1 3′UTR and miR-34a-5p are shown in Figure 5D. Overexpression of miR-34a-5p significantly suppressed the luciferase activity of the wild-type luciferase constructs but not the mutant ones in TPC-1 and BC-PAP cells (Figures 5E,F). Consistently, miR-34a-5p overexpression markedly repressed the mRNA and protein expression levels of TK1 in both TPC-1 and BC-PAP cells (Figures 5G–J).

## Discussion

Patients with advanced thyroid carcinoma had poor prognosis with low overall survival (13, 14). Unfortunately, the underlying mechanisms of thyroid carcinoma progression remain unclear. The elevated expression of TK1 has been implicated in the progression of thyroid carcinoma (11), while the role of TK1 in thyroid carcinoma progression has not been explored. In this study, we found that serum TK1 levels were markedly increased in the patients with thyroid nodules. Further online data mining showed that TK1 expression was upregulated in thyroid carcinoma tissues, and higher expression of TK1 was correlated with shorter DFS of patients with thyroid carcinoma. Silencing of TK1 suppressed cell proliferation, invasion, migration, and EMT and also induced cell apoptosis in the thyroid carcinoma cell lines. *In vivo* data showed that TK1 knockdown inhibited *in vivo* tumor growth of thyroid carcinoma cells. Moreover, the upregulated TK1 in the thyroid carcinoma cells was associated with the downregulation of miR-34a-5p.

The prognostic role of TK1 has been widely studied in the cancer studies (5, 6, 15, 16). The increased Tk1 mRNA levels in the plasma-derived exosomes are associated with clinical resistance to CDK4/6 inhibitors in metastatic breast cancer patients (17). Using immunohistochemistry, TK1 was found to be located on the cellular membrane of the colorectal, lung, and breast cancer cells. The upregulation of TK1 in these malignant tissues indicated the potential prognostic role of TK1 (18). Serum detection of TK1 is sensitive and specific for the prediction of early stage and advanced lung cancer (19). In this study, it was showed that serum TK1 levels were upregulated in patients with thyroid nodules, suggesting that TK1 might be involved in the development of thyroid cancer. Our findings were in accordance with the previous studies that thyroid carcinoma tissues were accompanied with higher expression levels of TK1 (20). Besides, the i*n vitro* studies showed the consistent upregulation of TK1 in thyroid carcinoma cells.

In this study, it was the first time to show that TK1 knockdown suppressed *in vitro* and *in vivo* carcinoma cell progression. It was reported in pancreatic cancer the silencing of TK1 suppressed cancer cell proliferation via inducing S phase arrest by P21 upregulation. P21was an inhibitor of the cyclin-dependent kinase and could inhibit the activation of CDK1, CDK2, and CDK4. TK1 might interact with P21 by combining with the C-terminal domain of P21, and promote the proliferation of cancer cells (21). In addition, growth and differentiation factor 15 was considered to be the main downstream mediator of TK1 function, which induced the metastatic attributes of lung cancer cells (22). Taken together, the results in this study might imply that TK1 promoted thyroid carcinoma progression via P21 or growth and differentiation factor 15 by increasing cell proliferation, invasion, and migration.

The bioinformatic prediction results revealed that TK1 could be potentially regulated by miRNAs. MiR-34a-5p was found to be upregulated in the thyroid carcinoma cell lines. Further studies showed that miR-34a-5p repressed TK1 expression in thyroid carcinoma cells via targeting the 3′UTR of TK1. Previously, miR-34a-5p was shown to be downregulated in the thyroid carcinoma tissues and played a tumor-suppressive role in the thyroid carcinoma cells (23). In addition, miR-34a-5p also functioned as tumor-suppressive factor in esophageal squamous cell carcinoma (24), non-small cell lung cancer (25), and breast cancer (26). Collectively, the upregulation of TK1 may be related to downregulated miR-34a-5p expression, which contributed to the enhanced progression of thyroid carcinoma.

In summary, the results in this study demonstrated the upregulation of TK1 in thyroid nodules as well as thyroid carcinoma tissues. The downregulated miR-34a-5p in thyroid carcinoma resulted in higher TK1 level, which promoted thyroid carcinoma cell proliferation, invasion, and migration. This study may provide novel insights into the role TK1 played in regulating thyroid carcinoma progression. However, there are still some limitations in this study. For example, although higher level of TK1 was confirmed in thyroid cancer patients, the lower expression level of miR-34a-5p was not detected. In addition, the upregulated TK1 in thyroid cancer patient may be resulted from the downregulated miR-34a-5p, but the upstream and downstream molecular mechanisms were still unclear. To further investigate and elucidate the role of TK1 in thyroid carcinoma progression, future studies are necessary.

## Data Availability Statement

The raw data supporting the conclusions of this article will be made available by the authors, without undue reservation, to any qualified researcher.

## Ethics Statement

The studies involving human participants were reviewed and approved, and all the experimental protocols were approved by the Ethics Committee of the First Affiliated Hospital of Southern University of Science and Technology. The patients/participants provided their written informed consent to participate in this study. The animal study was reviewed and approved by All the animal experiments were approved by the Animal Ethics Committee of First Affiliated Hospital of Southern University of Science and Technology.

## Author Contributions

WW, GW, and FD designed and supervised the whole study. CL, JW, and LZ performed the experiments. HH, PZ, and ZP collected the serum samples. FL and JC performed the data analysis. WW wrote the manuscript. All authors approved the manuscript for submission.

### Conflict of Interest

The authors declare that the research was conducted in the absence of any commercial or financial relationships that could be construed as a potential conflict of interest.
